# Environmental Dependence of Genetic Constraint

**DOI:** 10.1371/journal.pgen.1003580

**Published:** 2013-06-27

**Authors:** Marjon G. J. de Vos, Frank J. Poelwijk, Nico Battich, Joseph D. T. Ndika, Sander J. Tans

**Affiliations:** FOM Institute AMOLF, Amsterdam, the Netherlands; Harvard University, United States of America

## Abstract

The epistatic interactions that underlie evolutionary constraint have mainly been studied for constant external conditions. However, environmental changes may modulate epistasis and hence affect genetic constraints. Here we investigate genetic constraints in the adaptive evolution of a novel regulatory function in variable environments, using the *lac* repressor, LacI, as a model system. We have systematically reconstructed mutational trajectories from wild type LacI to three different variants that each exhibit an inverse response to the inducing ligand IPTG, and analyzed the higher-order interactions between genetic and environmental changes. We find epistasis to depend strongly on the environment. As a result, mutational steps essential to inversion but inaccessible by positive selection in one environment, become accessible in another. We present a graphical method to analyze the observed complex higher-order interactions between multiple mutations and environmental change, and show how the interactions can be explained by a combination of mutational effects on allostery and thermodynamic stability. This dependency of genetic constraint on the environment should fundamentally affect evolutionary dynamics and affects the interpretation of phylogenetic data.

## Author Summary

Epistatic interactions limit the number of adaptive trajectories to peaks on evolutionary fitness landscapes, and may therefore hamper the progress of evolution. Recent research has focused on adaptive landscapes in one constant environment. However, adaptive evolution is generally known to occur in variable, heterogeneous environments. Here, we have constructed fitness landscapes of three inverse *lac* repressor variants in two contrasting environments. We find that the epistatic interactions between the pairs of mutations are profoundly altered upon an environmental change. We develop a new graphical method to analyze the underlying higher-order interactions between genetic changes and the environment, and explain the complex environmental dependencies in terms of simple molecular mechanisms. Our results show that the information about epistatic interactions acquired in one environment does not inform on the true limitations of adaptive evolution. We argue that this dependency of genetic constraints on the environment will have important effects on the progress of adaptation in heterogeneous environments, and will affect our ability to establish realistic genealogies from the phylogenic record.

## Introduction

As pointed out by Sewall Wright in the 1930's, the genetic makeup of a biological system should determine not only current functionality but also affect future evolutionary change [1]. How the present genetic architecture constrains future adaptive evolution is now starting to be addressed experimentally [2]–[4]. By systematically constructing single-mutant neighbors and assaying their function or fitness, proteins ranging from TEM β-lactamase [3] to steroid receptors [5] have been shown to exhibit sign epistasis, in which one mutation can be beneficial or deleterious depending on the presence of another mutation. Sign epistasis by itself does not imply evolutionary constraint, as the interacting mutations may simply not play a role in adaptation. However, when mutations essential for functional innovation exhibit sign-epistasis, constraints emerge for evolutionary trajectories that depend on fixing one adaptive mutation after another by positive selection [6]. For sign-epistatic interactions, the number of such adaptive trajectories is reduced. Two mutations may also be deleterious individually but jointly beneficial, as observed for mutations in the regulator MTH1 and glucose transporters HXT6/HXT7 in *Saccharomyces cerevisiae* [7] and between *argH12* and *pyrA5* mutants leading to arginine and pyrimidine deficiency in *Aspergillus niger* [8]. Such reciprocal sign epistasis is a necessary condition for multiple peaks in the fitness landscape [9], which can completely block evolutionary trajectories in which mutations are fixed one-by-one by positive selection. Because of this ability to arrest, delay, and divert evolution, genetic interactions have been speculated to play a central role [10] in speciation [11], [12], the maintenance of biodiversity [13], and developmental evolution [14], [15].

So far, epistastic interactions have been studied predominantly for environments that are constant in time and favor a single function or phenotype. However, natural environments are characterized by irregular temporal changes, which in turn impose temporally changing demands on the expressed phenotypes. Indeed, the complexity of regulatory systems is considered to have evolved in response to environmental heterogeneity [16], [17]. Experimentally, mutations are commonly observed to have different effects in different environments [18]–[20]. For example, in *Escherichia coli* the fitness effects of single Tn10 transposon insertion mutations [21]and mutations conferring resistance to bacteriophages λ and T4 have been shown to depend on the genetic background and the environment [22]. Correlations exist between epistatic interactions in plant viruses and their hosts [23], and trade-offs have been observed between the effect of mutations in the presence of certain types or concentrations of antibiotics in *Escherichia coli* [24], [25] and *Pseudomonas aeruginosa* [26].

These observations raise the question to which extent constraints themselves change when the environment changes. If mutations essential to functional innovation exhibit sign-epistatic interactions that are modulated by environmental change, adaptive trajectories will be drastically affected. For instance, evolutionary change hampered by adaptive valleys in one environment could be opened up to positive selection in another. Conversely, trajectories that can be positively selected for in constant environments [2], [3] could be blocked by environment-induced sign epistasis, which could slow down overall evolutionary progress or drive adaptation to dead ends in genotype space. This environmental control over the accessibility of adaptive trajectories goes beyond merely defining a variable selective environment, and would invalidate commonly held assumptions in analyzing the historical evolutionary record by phylogenetic reconstruction (23).

These elementary issues can be readily investigated using a simple phenotype that responds to the environment. We focused on one of the most well-understood model systems for environmentally controlled gene expression, the *Escherichia coli lac* repressor LacI [27]. We considered the evolutionary transition to a variant that exhibits an altered regulatory response [28]. In the presence of the wild-type repressor, LacI_wt_, the *lac* operon is induced by the ligand IPTG, whereas in the presence of the variant LacI_inv_, expression is suppressed by IPTG. We have previously isolated LacI variants with such inverse phenotypes in evolutionary experiments [28] (Text S1), which serve as a basis to systematically assess how the environment affects epistasis between the mutations required for inversion. We find that the epistasis is highly environment-dependent, which implies that epistasis perceived in a constant environment does not properly inform on the evolutionary constraints in a variable environment. We can explain the generic pattern of higher-order genotype x genotype x environment interactions that is observed in all three variants using a simple model of changes in the allosteric transition and in protein stability.

## Results

### Environmental dependence of epistasis

**Figure 1:**
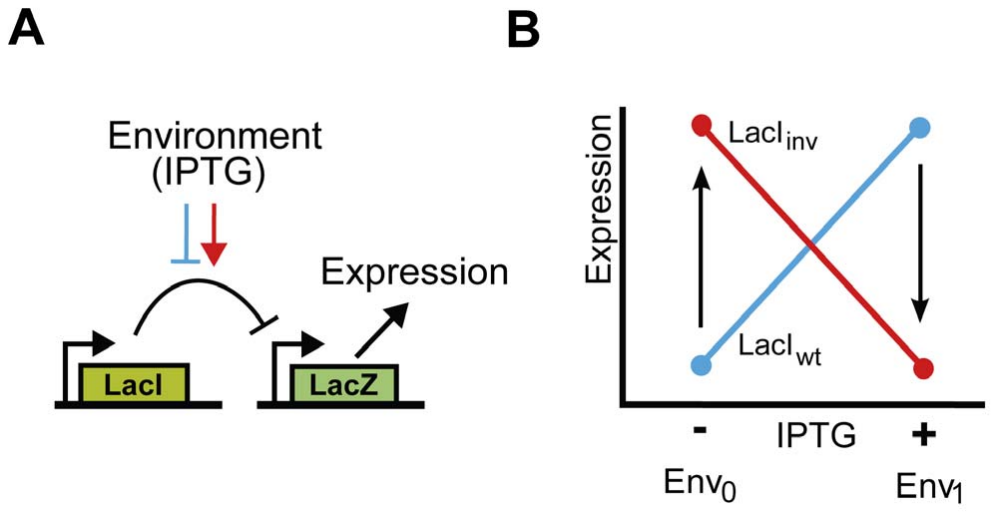
Functional description and schematic representation of genetic variants in the *lac* system. A) Schematic representation of the genetic system in *E. coli*. The *lac* repressor, LacI, controls expression of LacZ. The system responds to IPTG. IPTG acts as an inducer in the wild type LacI (blue block-arrow), and as a co-repressor in the phenotypically inverse mutants (red arrow). B) Environmental dependence of the expression level of lacZ. Expression levels are measured in two environments. For the wild type LacI (LacI_wt_), LacZ expression level is high in the presence of IPTG (Env_1_) and low in its absence (Env_0_) (blue line). For the inverse LacI variant (LacI_inv_), LacZ expression level is high in the absence of IPTG (Env_0_) and low in its presence (Env_1_) (red line). We consider mutational trajectories from the wild type to the inverse variant (arrows).

To investigate the interplay between the environment and epistasis we focused on three inverse LacI variants [28] (Text S1). The three inverse variants each contained three to six point mutations relative to LacI_wt_. For all variants, three mutations appeared essential for the inverse function, as was determined by engineering *lacI* variants that contained sub-sets of these mutations. We denote these three inverse variants as LacI_inv1_ (S97P, R207L, T258A), LacI_inv2_ (S97P, L307H, L349P) and LacI_inv3_ (S97P, G315D, P339H). Note that all share the mutation S97P. Next, we constructed all the single and double mutants, and assayed the operon expression phenotypes in the absence of IPTG (Env_0_) and in the presence of 1 mM IPTG (Env_1_) (Table S1) using a fluorogenic reporter assay (materials and methods) (Figure 1A). Given the evolutionary objective of inversion, a high operon expression level is favored in Env_0_, whereas a low expression level is favored in Env_1_ [28] (Figure 1B).

To compare the epistasis in each environment, we classified the epistatic (genotype x genotype) interactions for all pairs of mutations for each of the three inverse LacI variants. We distinguished three categories: magnitude epistasis (M) - both mutations are either beneficial or deleterious, irrespective of the genetic background, sign epistasis (S) – the effect of one mutation changes sign depending on the genetic background, or reciprocal sign epistasis (R) - both mutations are individually deleterious, but beneficial in combination [4]. Neutral mutations are not positively selected and are thus grouped under deleterious. We find that nine out of the eighteen mutation pairs display the same category in environments Env_0_ and Env_1_ (Table 1). For instance, in the P349 background, L307H and S97P exhibit sign epistasis in both environments (Table 1, LacI_inv2_). Note that for all these nine pairs, the *magnitude* of the mutational effect does depend on the environment, but the sign does not. For the other nine mutation pairs, the category of epistasis differs between the two environments (Table 1). Some sign epistatic interactions are switched ‘off’ by the addition of IPTG. In the P97 background for instance, IPTG induces a sign change in the effect of R207L; it transforms the sign-epistasis between R207L and T258A in Env_0_ to magnitude epistasis in Env_1_ (Table 1, LacI_inv1_). Sign epistasis is turned ‘on’ between other mutations. For instance, in a P97 background, L349P and L307H exhibit sign epistasis in an environment without IPTG, and reciprocal sign epistasis with IPTG (Table 1, LacI_inv2_). Thus, environmental signals modulate sign-epistatic interactions between residues involved in the functional inversion of LacI.

**Table 1:** Genetic interactions and their environmental dependence.

Genetic interaction			LacI_inv1_		Genetic interaction			LacI_inv2_		Genetic interaction			LacI_inv3_	
S97P	R207L	T258A	Env_0_	Env_1_	S97P	L307H	L349P	Env_0_	Env_1_	S97P	G315D	P339H	Env_0_	Env_1_
X	X	○	M	M	X	X	○	S(L307H)	M	X	X	○	S(G315D)	S(G315D)
X	○	X	S(T258A)	M	X	○	X	S(L349P)	M	X	○	X	M	M
•	X	X	S(R207L)	M	•	X	X	S(L349P)	R	•	X	X	S(P339H)	M
X	•	X	S(T258A)	M	X	•	X	M	S(L349P)	X	•	X	S(P339H)	M
○	X	X	M	M	○	X	X	M	M	○	X	X	M	M
X	X	•	M	M	X	X	•	S(L307H)	S(L307H)	X	X	•	S(G315D)	S(G315D)

The genetic interactions are indicated for three inverse LacI variants. Each row details the interactions between two mutations, each indicated by an X, either in a LacI_wt_ background (denoted by a ○), or a single mutant background (denoted by a •). We consider three types of interactions: M, magnitude epistasis; S, sign epistasis; R, reciprocal sign epistasis. The mutation that changes sign is indicated between brackets. The data shows that most genetic interactions display different types of epistasis in each of the two environments. The significance of the phenotypic effect of mutations in LacI is tested with a *t*-test in conjunction with a Bonferroni correction for multiple comparisons (*P*<0.05).

### Genotype x genotype x environment interactions

**Figure 2:**
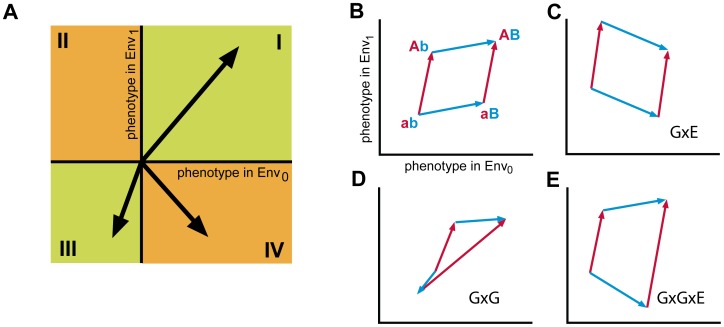
Analysis of higher order genotype-environment interactions. A) Schematic representation of the effect of mutations on phenotype in two environments. Mutations are represented as vectors with the start in the origin of the coordinate system. Mutations are either beneficial in both environments, Env_0_ and Env_1_ (quadrant I), beneficial in one environment but deleterious in the other (quadrant II or IV) or deleterious in both environments (quadrant III). Classification of interactions between two mutations in two environments: B) Opposite sides of the polygon represent the same mutation in different genetic backgrounds (a to A (red) in background b or B, and b to B in background a or A (blue)). Absence of epistasis or genotype x environment (GxE) interactions. The vectors of opposing sides are positioned in either quadrant I or III, and the polygon is a simple parallelogram, in the absence of magnitude epistasis. C) Genotype x environment interactions. Opposing sides of the parallelogram are located in the same quadrant. At least one pair of opposing sides lies in quadrant II or IV. D) Sign epistasis. Here, mutation b to B changes sign depending on the genetic background (a or A) in both environments. E) Higher-order GxGxE interactions. At least one pair of vectors from opposing sides of the polygon are located in different quadrants of which at least one vector is located in quadrant II or IV. Note however, that the presence of both GxE and GxG interactions not necessarily implies the presence of GxGxE interactions. In the case that one mutation displays sign epistasis, and the other mutation GxE, their combination does not imply GxGxE (Figure S1).

The above classification of genetic interactions into categories reveals a dependence on the environment, but it does not offer intuitive insights into their causes. These dependencies may also be viewed as three-way interactions between two genetic changes and one environmental change. Hence, they can be denoted as genotype x genotype x environment interactions, or briefly GxGxE; analogous to two-way GxG interactions between two genetic changes in a single environment, or two-way GxE interactions between one genetic change and one environmental change [17]. To analyze these higher-order interactions, we introduced a graphical method (Figure 2A). Mutations are represented as vectors in a two-dimensional coordinate system, where the axes indicate the corresponding changes in expression phenotype in both environments. A vector pointing to quadrant I signifies functional improvements in both environments, whereas quadrants II and IV denote improvement in one environment and deterioration in the other, and quadrant III denotes deterioration in both. The probability of fixing neutral mutations is low compared to positively selected mutations that confer functional improvements [6], [29]. Mutations that are neutral in both environments therefore correspond to quadrant III, while mutations that are neutral in one environment and beneficial in the other correspond to quadrants II or IV. Thus, mutations in quadrants II and IV indicate sign-changing GxE interactions.

Higher-order interactions between two or more mutations and the environment can be visualized by sets of paths composed of two or more mutational vectors (Figure 2). The two mutational paths from genotype ab to AB (*via* Ab or *via* aB) form a four-sided polygon. The polygon is a simple parallelogram in the absence of any genetic interactions, which may occur either without (Figure 2B) or with GxE interactions (Figure 2C). Deviations from the parallelogram indicate genetic interactions, or epistasis. Vectors at opposing sides of the polygon that have different angles but point in the same quadrant indicate magnitude epistasis. Opposing vectors pointing in different quadrants indicate sign-epistatic interactions (GxG, Figure 2D), and when the sign change of opposing vectors is conditional on the environment higher-order GxGxE interactions can be observed (GxGxE, Figure 2E). Thus, higher-order interactions between mutations and the environment can be graphically recognized and classified using the mutational vector plots.

### Generality of the interactions

**Figure 3:**
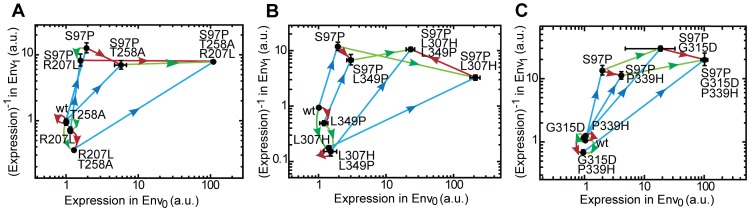
Adaptive trajectories towards the three inverse LacI variants. The three inverse LacI variants all contain three mutations. Each mutation is represented by a vector (see Figure 2). The axes indicate expression without IPTG in Env_0_ and expression with IPTG in Env_1_. Expression levels in both environments are normalized to the LacI_wt_ level. Note that expression along the vertical axis is represented as (Expression)^−1^, as during inversion the expression level in Env_1_ decreases. The inverse, triple mutant, is located in the upper right corner of the plot. A) LacI_inv1_: S97P (blue), R207L (green), T258A (red). B) LacI_inv2_: S97P (blue), L307H (green), L349P (red). C) LacI_inv3_: S97P (blue), G315D (green), P339H (red). The significance of the phenotypic effect of mutations is tested with a *t*-test with Bonferroni correction for multiple comparisons (*P*<0.05), error-bars are standard deviations, n = 3.

We analyzed the interactions for the three LacI variants by displaying the expression data as mutational vectors in Figure 3A, B and C. Because the transition to inversion is characterized by a decreasing operon expression in the presence of IPTG (Env_1_) and an increasing operon expression in the absence of IPTG (Env_0_), we plotted 1/expression in Env_1_ against the expression in Env_0_, such that the closer the phenotype comes to the objective of inversion, the more it moves towards the upper-right corner of Figure 3.

Inspection of the polygon shapes shows that half (50%) lack the signatures of sign-changing higher-order interactions involving mutation pairs and the environment. For instance in Figure 3C, the opposing red and green vectors in the P97 background point in the same quadrant. The polygon is tilted, with both red vectors pointing in quadrant IV, indicating GxE interactions. However, the other half of the opposing mutational vector pairs in the polygons do not point in the same quadrant, indicating the pervasive presence of higher-order GxGxE interactions. For instance, in the P97 background, the addition of T258A turns the green vector (R207L) from quadrant III to IV, which is caused by the fact that R207L is neutral in the presence of IPTG and the absence of T258A, but increases expression by 20-fold in T258A's presence (Fig. 3A). Another example is the addition of L307H, which rotates the red vector (L349P) from quadrant IV to II in the P97 background, which indicates that the effect of L349P on expression changes sign in both environments due to L307H (Figure 3B).

Overall, the pattern displayed by the three variants in the vector plots (Figure 3A, B and C) is strikingly similar, in contrast to the diverse environmental dependence of epistasis seen in Table 1. The blue vectors initially point predominantly up along the Env_1_ axis (the expression level decreases with IPTG), as the expression level in Env_1_ is strongly decreased, but turn diagonally to the upper-right corner when the red and green mutations are added (the expression level increases simultaneously in the absence of IPTG) (Figure 3). On the other hand, the green and red vectors either point downward along the Env_1_-axis, (expression mainly increases in the presence of IPTG), or to the right along the Env_0_-axis (expression increases in the absence of IPTG). Mutation S97P appears responsible for this rotation of the red and green vectors: in the LacI_wt_ background they point along Env_1_, while in the P97 background they point along Env_0_. In other words, S97P represents a ‘switch’ that changes the interaction of the red and green mutations with the environment. This pattern is identical for all three inverse genotypes; all show a roughly similar rotation for the blue as well as for the red and green vectors. Thus, while the genetic solutions to the phenotypic inversion are different in the three variants, the main features of the underlying map of the interactions between genotypes and the environment are general.

Note that one may also consider the presence of higher-order interactions that are purely genetic. Specifically, such GxGxG interactions arise when the addition of a third mutation changes the category of the two-way epistatic motif. For instance, in the wild type background, both green (L307H) and red (L349P) vectors point downward or are neutral along the Env_1_ axis (Figure 3B), and hence point to magnitude epistasis. However, upon the application of S97P (Figure 3B, blue vectors), one green and one red vector still points down, but one green and one red vector is rotated upwards. Thus, L307H and L349P display reciprocal sign epistasis in the presence of P97, and hence their three-way interaction in Env_1_ cannot be captured by two-way epistasis alone. Note that this GxGxG interaction itself may in turn be dependent on the environment, indicating GxGxGxE interactions. Among other things, the presence of higher-order genetic interactions illustrates that conclusions on the accessibility of a genotype must be carefully considered. This is particularly relevant when it is unclear to what extent the mapped genotype space fully determines the considered function, as an untested mutation could open up mutational pathways to selection, which otherwise may have been considered blocked [30]. The principle of such effects of higher-order genetic interactions have previously been captured [3], [4], [7], [15], [31] when mapping a larger landscape and assessing the mutational pathways within it. Nonetheless, the explicit presence of GxGxG interactions underscores the care that must be taken when formulating conclusions about selection and constraint from fitness landscapes.

The results also underscore that mechanisms that are comparatively simple on the molecular level, can give rise to GxE interactions. For instance, in the P97 background, L307H has the simple mechanistic effect of generally increasing expression both in the presence and absence of IPTG. In terms of selection, this change is beneficial in one environment (in the absence of IPTG), and deleterious in the other (in the presence of IPTG). Hence, L307H gives rise to a GxE interaction, a trade-off. Given the generic purpose of regulatory functions to modulate biological functions in response to input signals, one can expect such trade-offs that originate from simple molecular mechanisms to be rather generally present.

### Molecular basis of the interactions

The observed generality of the genotype-environment interaction maps (Figure 3) suggests that they result from a generic structural cause. However, the positions of the mutated residues within the LacI crystal structure do not directly reveal generic features, as they appear scattered throughout the structure, with different locations for the different variants (Figure S2). Also, the mutations are not positioned at obvious functional sites such as the DNA or ligand binding regions. Alternatively, the origin of the interactions may be rooted in the mechanism of inversion, which has been speculated to be based on two effects [28], [32]. First, the allosteric transition from high to low operator affinity is thought to be impeded by S97P, as P97 cannot form the transient bond with K84′ and V94′ [33], which in turn locks the structure in the DNA-bound confirmation [34], [35]. Second, the response to inducer is assumed to be inverted through changes in the thermodynamic stability of the protein: the additional two mutations in each variant would lower the stability in the absence of IPTG, which would confer an increased expression level in Env_0_, while the binding of the ligand IPTG to LacI would confer a stabilizing effect that conserves a low expression level in Env_1_. Our experiments showed that in a LacI_wt_ background, S79P lowers expression in Env_1_ to repressed levels while maintaining a relatively low expression level in Env_0_. Thus, these data are indeed consistent with the proposed locking of LacI in the DNA-bound confirmation.

**Figure 4:**
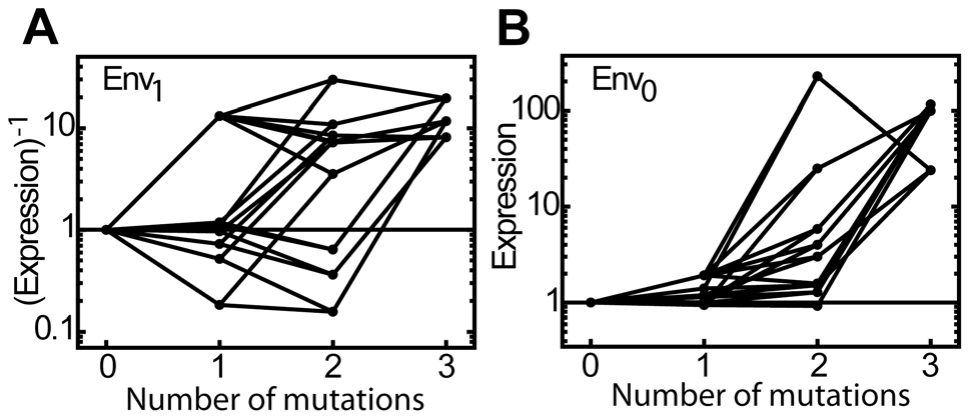
Mutational effects on expression in both environments. Expression along mutational trajectories towards all three LacI_inv_ variants. A) (Expression)^−1^ in Env_1_ along all trajectories. B) Expression in Env_0_ along all trajectories. For all three inverse variants, expression in Env_0_ increases for nearly all mutational steps, in contrast to the more erratic pattern in Env_1_ .

The data further show that expression in Env_1_ varies along the mutational trajectories from LacI_wt_ to LacI_inv_ (Figure 4A). In contrast, in Env_0_, the trajectories to inversion show a generic increasing trend in the expression level; all first mutations yield little to no changes, while second and third show increasingly large expression increases (Figure 4B). The pattern of changes in expression level in both environments is consistent with stability-decreasing mutations, as: 1) correlation between the stability and the expression level should be stronger in Env_0_, as the ability to tightly bind DNA in that environment is dependent on structural stability, in contrast with the ability to efficiently release from the DNA in Env_1_, and 2) it has been argued that protein function is robust against initial stability decreases, but can be expected to deteriorate when accumulated mutations drive the system across their so-called stability threshold [36]–[38]. We investigated the destabilizing effect of the mutations by analyzing the stability changes due to amino acid substitutions *in silico* with FoldX [39], [40]. In the absence of IPTG (Env_0_), FoldX indeed showed significant stability decreases for most (8 out of 11, Table S2) of the studied mutants, including S97P. The expression measurements suggest that in particular S97P brings LacI to the edge of the stability threshold, as subsequent mutations strongly increase expression (Figure 3, Env_0_). Thus the S97P substitution acts as a switch that systematically alters the phenotypic effect of the other mutations.

While we have addressed the central features of the interaction map, various more detailed interactions between mutations and the environment remain to be explained mechanistically. However, overall the analysis indicates that the combined effects of two independent and simple molecular mechanisms can explain complex higher-order GxGxE interactions between multiple mutations and the environment.

## Discussion

Recent systematic reconstructions of evolutionary intermediates have provided a first view on adaptive landscapes and the causes of evolutionary constraint [4]. Sign epistatic interactions between mutations have been shown to limit the number of mutational trajectories that can be followed under positive selection in constant environments [2], [3]. Directed evolution experiments revealed evolutionary constraints that delay or prevent adaptation [15], [28], and measured trade-offs between environments indicated how such constraints affect selection in variable environments [28], [41]–[43]. Here we investigated how the environment affected the adaptive landscape describing a specific functional innovation, by reconstructing the evolutionary intermediates on route to three different inverse LacI genotypes.

The three evolved genotypes indicated a redundancy within the LacI genetic architecture to develop regulatory functions that respond to the environment, mirroring similar results obtained for microbial populations evolving in constant environments [44]–[46]. We found that a mechanistic model of inversion provided an explanation for the origin of this parallelism. First, a mutation (S97P) blocks the IPTG-induced allosteric transition, and thus affects expression only in the presence of IPTG. Second, the initial mutations have little effect on the ability to repress in the absence of IPTG, while later mutations have a large effect. Third, binding to the ligand IPTG increases the protein stability and hence the ability to repress. Thus, a combination of simple molecular mechanisms can explain the observed complex higher-order interactions between multiple genetic changes and an environmental change.

The data showed that the genetic epistasis in LacI was pervasively dependent on the environment. As the studied genetic changes were not chosen randomly but jointly confer a novel regulatory response, these results inform on constraints in the evolution of a novel biological function. They indicate that limitations in the selective accessibility of trajectories, as detected in a constant environment, not properly inform on evolutionary limitations in the natural variable environment. Due to the environmental dependence of epistasis, some trajectories are closed-off by environmental change while others are opened-up to positive selection. Intriguingly, a consequence of environmental dependence of epistasis is that few mutations are blocked in all environments, and many are positively selected in at least one environment. This suggests that genetic constraints may be more readily overcome in certain variable environments than expected from epistasis detected in constant environments [47], [48].

More generally, the results underscore the complex and diverse roles of the environment in evolutionary dynamics. The environment does not only define a selective pressure on a phenotypic trait or induce a phenotypic change, but also modulates the underlying genetic constraint. This interdependence has a number of consequences. For instance, it affects our ability to understand the evolutionary record as interpreted from extant genetic sequence data. By modulating evolutionary constraint in time, environmental variations can change substitution rates across evolutionary trees [49], [50], referred to as heterotachy, even if selection on a phenotypic trait is constant. It can result in topological inaccuracies in phylogenetic trees [51] such as long-branch biases [52], [53] and a lack of phylogenetic resolution [52], [54] if the underlying adaptive landscapes are shaped differently in each of the environments. This can ultimately affect the predictive power of phylogenetic reconstruction techniques in their use for the prognosis of the emergence and the spread of diseases, such as the spread of the influenza virus [55], where the host can be viewed as a biotic environment [56]. And lastly, it renders a walk on evolutionary branches of life unpredictable and unrepeatable [3], [57], as some adaptive trajectories are constrained in some environments, but not in others.

It will be intriguing to explore the prevalence of the higher-order genotype x genotype x environment interactions in other biological systems. It is not obvious that all biological functions will show such interactions; in particular those specialized to a single environmental factor. On the other hand, the ability to respond to environmental stimuli is one of the defining properties of living systems. Given the inherent interdependency between regulatory systems and the environment, we expect that such insights into the interplay between genetic architecture and the environment will be crucial for a mechanistic understanding of the evolution of biological functions.

## Materials and Methods

### Strains

*Escherichia coli* K12 strain MC1061 [58], which carries a deletion of the *lac* operon was used in all experiments. This strain was obtained from Avidity LLC, Denver CO, USA, as electrocompetent strain EVB100 (containing an additional chromosomal *birA*). Plasmid pRD007 was constructed based on the pZ vector system [59] and contains LacI, driven by the P_L_O1-Tet promoter. The reporter plasmid pReplacZ, used for the quantification of LacZ expression, was created by deletion of *lacI* and Ptrc in pTrc99A [60] followed by insertion of the Plac-lacZ fragment of MG1655 [61].

### Media

In all experiments EZ defined rich medium (Teknova, Hollister, CA, USA) with 0.2% glucose and 1 mM thiamine HCL (Sigma) was used. Isopropyl β-D-1-thiogalactopyranoside (IPTG) was purchased from Sigma, and was added to the medium, if applicable, in a 1 mM quantity.

### Reconstruction of (intermediate) mutants

Mutations were introduced into the coding region of *lacI* by site-directed mutagenesis with the QuickChange II–E Site–Directed Mutagenesis Kit (Stratagene, USA) according to the manufacturer's protocol [28]. Constructs are available upon request.

### Expression measurements

Cultures were grown at 37°C in a Perkin & Elmer Victor^3^ plate reader, at 200 µl per well in a black clear-bottom 96 well plate (NUNC 165305). Expression measurements were performed in EZ Rich Defined medium with added 0.2% glucose (Teknova, Hollister, CA, USA, cat. nr. M2105) supplemented with 1 mM thiamine HCl and the appropriate antibiotics for the selective maintenance of plasmid pRD007 and pRepLacZ. Optical density at 600 nm was recorded every 4 min, and every 29 min 9 µl sterile water was added to each well to counteract evaporation. When not measuring, the plate reader was shaking the plate at double orbit with a diameter of 2 mm. Cells were fixed after the cultures had reached an optical density of at least 0.015 and at most 0.07, by adding 20 µl FDG-fixation solution (109 µM fluorescein di-β-D-galactopyranoside (FDG, Enzo Life sciences, NL), 0.15% formaldehyde, and 0.04% DMSO in water). Fluorescence development was measured every 8 min (exc. 480 nm, em. 535 nm), as well as the OD_600_. Shaking and dispensing conditions were as mentioned above. When cells are not induced with IPTG, directly before or after fixation an appropriate amount of inhibitive IPTG was added. Analysis of the fluorescence trace is as described in [28].

### Statistical analysis

Significance of the phenotypic effect of mutations in LacI was tested with a *t*-test with Bonferroni correction for multiple comparisons (*P*<0.05). While the phenotypic effect of S97P in the wild type background in Env_0_, was not significant in the data set of one inverse Lac variant (LacI_inv3_), it was significant for the two other variants, and hence S97P was considered significant for the wild type background and Env_0_.

### FoldX stability analysis

A FoldX plugin [40](version 1.4.22) in the Yasara software package [62](version 11.11.4) was used for the stability analysis of the single, double and triple (only LacI_inv1_) mutants on basis of the DNA bound dimeric LacI crystal structure (1EFA) [63], which lacks the tetramerization domain. The structure was minimized without ONPF before addition of the mutations, and the calculation of the stability changes. The stability calculation was performed three times for each mutation, with standard deviations among the calculations smaller than ΔΔG = 0.5 kcal/mol.

## Supporting Information

Figure S1 GxE and GxG is not sufficient for GxGxE. The presence of both genotype x genotype and genotype x environment interactions in one motif is not sufficient for genotype x genotype x environment interactions. If the effect of one mutation is affected by the genetic background, but not by the environment (a to A), and the other by the environment, but not the genetic background (b to B), then these mutations do not exhibit genotype x genotype x environment interactions.
(DOC)

Figure S2 Mutations from the three inverse variants mapped on the LacI crystal structure. The mutated amino acids of all three inverse variants are depicted as space filling residues in the wild type LacI structure. They are color coded on basis of their grouping (see Table S2). Note that the tetramerization domain is absent in this crystal structure. Red residue, involved in multimerisation of the protein. Green residues, located on or near the surface of the protein. Blue residue, involved in allosteric transition [33]. Since this dimeric structure lacks the tetramerization domain, residues P339 and L349, are not depicted. Mutations mapped on crystal structure 1EFA (PDB) [63].
(DOC)

Table S1 Expression level of genetic variants in two environments. The expression level of LacZ was measured in two environments by a fluorogenic reporter assay. Env_0_, in the absence of IPTG and Env_1,_ in the presence of 1 mM IPTG. Errors are standard deviations, n = 3.
(DOC)

Table S2 Changes in stability and location of mutations in the protein. Stability changes are calculated using the FoldX plugin in Yasara of the 1EFA crystal structure [63] (materials and methods). Positive changes in ΔG indicate destabilization of the protein, whereas negative changes in ΔG indicate a stabilization effect. The 1EFA crystal structure lacks the tetramerization domain. Therefore it was not possible to calculate the effect on stability induced by mutations located in the tetramerization domain of LacI_inv2_ (L349P) and LacI_inv3_ (P339H). The location of the mutations in the dimeric 1EFA crystal structure in LacI is depicted in Figure S1.
(DOC)

Text S1 The evolution of phenotypically inverse mutants.
(DOC)
