# Supplementary figures and images for: Environmental Dependence of Genetic Constraint
Source: PLoS Genet. 2013 Jun 27;9(6):e1003580. doi: 10.1371/journal.pgen.1003580 (PMC3694820; doi:10.1371/journal.pgen.1003580)

**Figure S1. GxE and GxG is not sufficient for GxGxE.**


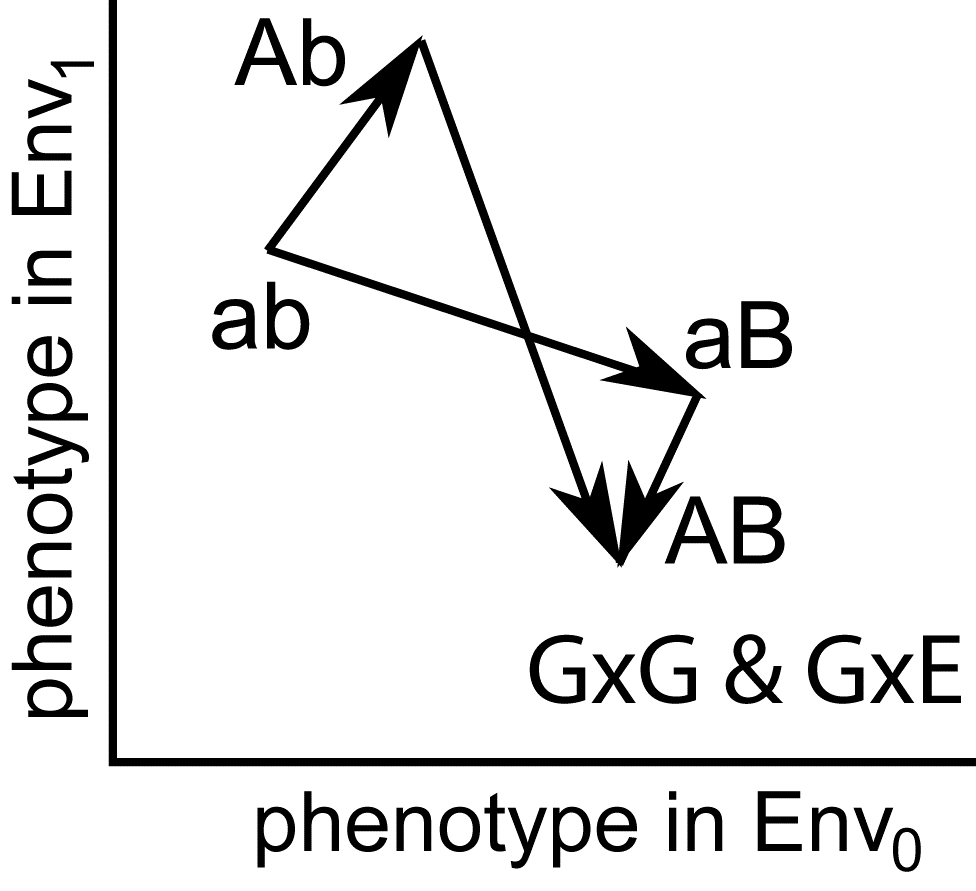

Supplement: Figure S1 — GxE and GxG is not sufficient for GxGxE. The presence of both genotype x genotype and genotype x environment interactions in one motif is not sufficient for genotype x genotype x environment interactions. If the effect of one mutation is affected by the genetic background, but not by the environment (a to A), and the other by the environment, but not the genetic background (b to B), then these mutations do not exhibit genotype x genotype x environment interactions. (DOC) [file pgen.1003580.s001.doc]

**Figure S2.** **Mutations from the three inverse variants mapped on the LacI crystal structure.**


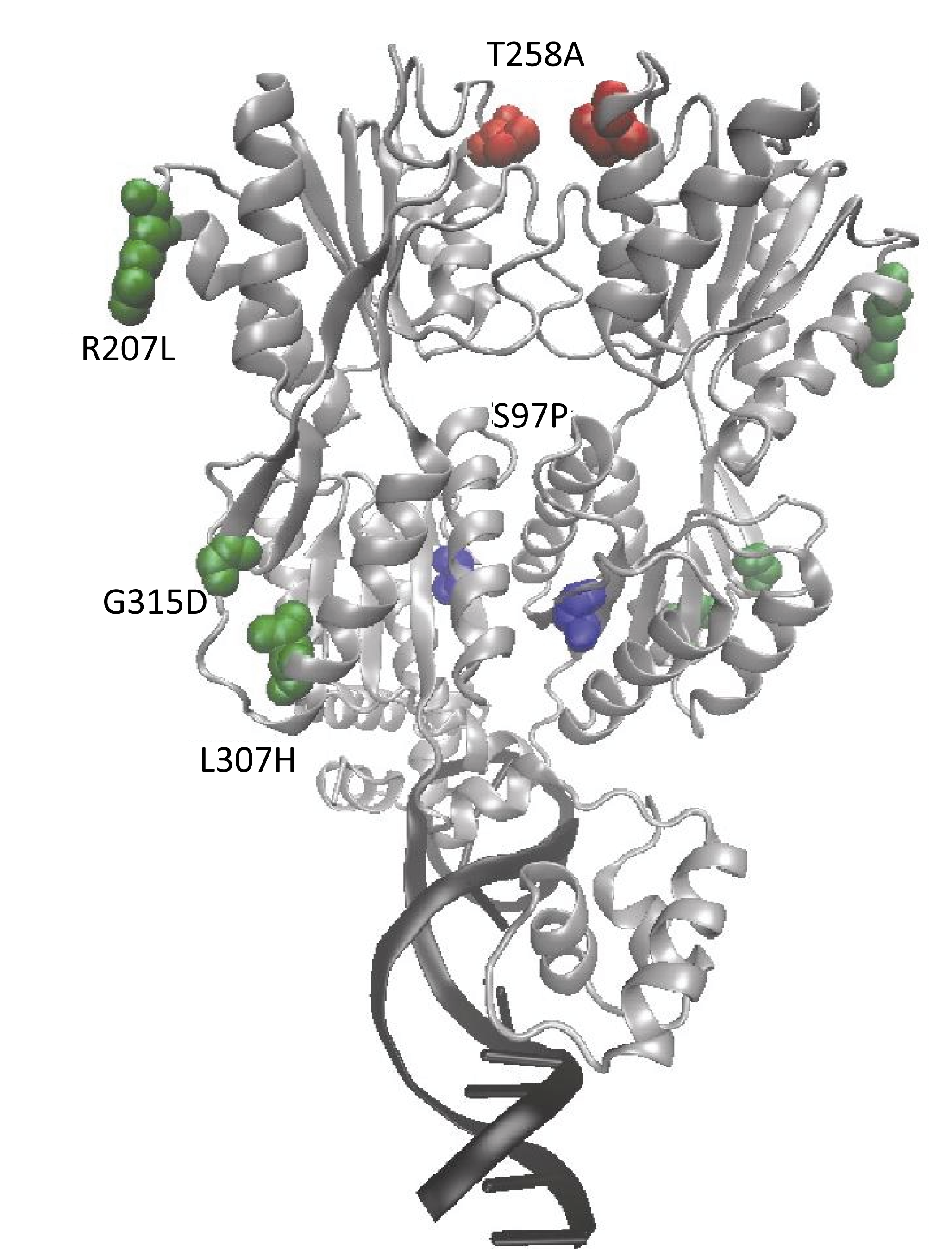

Supplement: Figure S2 — Mutations from the three inverse variants mapped on the LacI crystal structure. The mutated amino acids of all three inverse variants are depicted as space filling residues in the wild type LacI structure. They are color coded on basis of their grouping (see Table S2). Note that the tetramerization domain is absent in this crystal structure. Red residue, involved in multimerisation of the protein. Green residues, located on or near the surface of the protein. Blue residue, involved in allosteric transition [33]. Since this dimeric structure lacks the tetramerization domain, residues P339 and L349, are not depicted. Mutations mapped on crystal structure 1EFA (PDB) [63]. (DOC) [file pgen.1003580.s002.doc]
