# Supplementary material for: Environmental Dependence of Genetic Constraint
Source: PLoS Genet. 2013 Jun 27;9(6):e1003580. doi: 10.1371/journal.pgen.1003580 (PMC3694820; doi:10.1371/journal.pgen.1003580)
Supplement: Table S1 — Expression level of genetic variants in two environments. The expression level of LacZ was measured in two environments by a fluorogenic reporter assay. Env0, in the absence of IPTG and Env1, in the presence of 1 mM IPTG. Errors are standard deviations, n = 3. (DOC) [file pgen.1003580.s003.doc]

**Table S1. Expression level of genetic variants in two environments.**

| **Variant** | **Expression in Env_0_ (a.u.)** | | **Expression in Env_1_**  **(a.u.)** |  |
| --- | --- | --- | --- | --- |
| Wild type | 16 ± 0.5 | | 256 ± 12 |  |
| LacIinv1 |  | |  |  |
| R207L | 18.2 ± 1.4 | | 352 ± 39 |  |
| T258A | 15.9 ± 1.0 | | 264 ± 29 |  |
| R207L-T258A | 20.5 ± 0.2 | | 708 ± 43 |  |
| S97P | 30.7 ± 1.7 | | 19.4 ± 3.4 |  |
| S97P-R207L | 25.2 ± 1.3 | | 30.1 ± 6.3 |  |
| S97P-T258A | 93.5 ± 18 | | 25.3 ± 5.3 |  |
| S97P-T258A-R207L | 1846 ± 140 | | 31.4 ± 1.4 |  |
| Lacinv2 |  | |  |  |
| L307H | 22.4 ± 1.3 | | 1400 ± 122 |  |
| L349P | 19.1 ± 2.7 | | 494 ± 50 |  |
| L307H-L349P | 24.2 ± 5.3 | | 1633 ± 343 |  |
| S97P | 30.7 ± 1.7 | | 19.4 ± 3.4 |  |
| S97P-L307H | 3616 ± 624 | | 72.1 ± 7.9 |  |
| S97P-L349P | 48.2 ± 3.9 | | 34.4 ± 7.4 |  |
| S97P-L307H-L349P | 383 ± 56 | | 21.8 ±1.9 |  |
| LacIinv3 |  | |  |  |
| G315D | 15 ± 0.5 | | 232 ± 8.3 |  |
| P339H | 16.1 ± 1.0 | | 216 ± 6.0 |  |
| G315D-P339H | 14.7 ± 1.0 | | 399 ± 38 |  |
| S97P | 30.7 ± 1.7 | | 19.4 ± 3.4 |  |
| S97P-G315D | 399 ± 150 | | 8.60 ± 0.8 |  |
| S97P-P339H | 63.9 ± 2.2 | | 23.4 ± 3.2 |  |
| S97P-G315D-P339H | 1592 ± 208 | | 13.1 ± 3.0 |  |
|  |  | |  |  |
|  |  | |  |  |
|  | |  | |  |
