# Supplementary material for: Environmental Dependence of Genetic Constraint
Source: PLoS Genet. 2013 Jun 27;9(6):e1003580. doi: 10.1371/journal.pgen.1003580 (PMC3694820; doi:10.1371/journal.pgen.1003580)
Supplement: Table S2 — Changes in stability and location of mutations in the protein. Stability changes are calculated using the FoldX plugin in Yasara of the 1EFA crystal structure [63] (materials and methods). Positive changes in ΔG indicate destabilization of the protein, whereas negative changes in ΔG indicate a stabilization effect. The 1EFA crystal structure lacks the tetramerization domain. Therefore it was not possible to calculate the effect on stability induced by mutations located in the tetramerization domain of LacIinv2 (L349P) and LacIinv3 (P339H). The location of the mutations in the dimeric 1EFA crystal structure in LacI is depicted in Figure S1. (DOC) [file pgen.1003580.s004.doc]

**Table S2. Changes in stability and location of mutations in the protein.**

| **LacI_inv1_** | **ΔΔG kcal/mol** | **location in protein** |
| --- | --- | --- |
| T258A | -1.26 | dimerization domain |
| R207L | -1.04 | surface |
| S97P | 4.98 | core |
| R207L-T258A | -2.30 |  |
| S97P-R207L | 5.28 |  |
| S97P-T258A | 5.20 |  |
| S97P-R207L-T258A | 4.02 |  |
|  |  |  |
| **LacI_inv2_** |  |  |
| L349P |  | tetramerization domain |
| L307H | 4.40 | surface |
| S97P | 4.98 | core |
| L307H-L349P |  |  |
| L307H-S97P | 8.86 |  |
| S97P-L349P |  |  |
| L307H-L349P-S97P |  |  |
|  |  |  |
| **LacI_inv3_** |  |  |
| P339H |  | tetramerization domain |
| G315D | 5.12 | surface |
| S97P | 4.98 | core |
| G315D-P339H |  |  |
| S97P-G315D | 11.9 |  |
| S97P- P339H |  |  |
| S97P-G315D-P339H |  |  |
