# Supplementary material for: Environmental Dependence of Genetic Constraint
Source: PLoS Genet. 2013 Jun 27;9(6):e1003580. doi: 10.1371/journal.pgen.1003580 (PMC3694820; doi:10.1371/journal.pgen.1003580)
Supplement: Text S1 — The evolution of phenotypically inverse mutants. (DOC) [file pgen.1003580.s005.doc]

**Text S1. The evolution of phenotypically inverse mutants**

All three mutants described in this manuscript derived from an laboratory evolution experiment as described in . *Escherichia coli* MC1061 harboring a selective plasmid containing an operon with three genes *lacZ*, *sacB* and *cmR* whose expression is regulated by the *lac* repressor (*lacI*) experienced three rounds of laboratory evolution, in which the generation of mutations in *la*c*I* was separated from selection. Since only *lacI* was mutated, the genetic background remained wild type. Each round of evolution consisted of mutagenesis and selection. Mutations in *lacI* were produced by an error-prone polymerase (Stratagene Genemorph II Random Mutagenesis kit, Stratagene, USA) that generated on average three mutations per round. A strong selective pressure towards inversion of the regulatory function of LacI was applied by growing the cells for 6 hours in defined rich medium (Teknova, Hollister, CA, USA) supplemented with 1 mM thiamine-HCL (Sigma) containing 0.4% sucrose + 1mM IPTG (Sigma) followed by growth for 6 hours in 80 µg/ml chloramphenicol. The inverse mutants, of which the three LacI_inv_ derive, were sequenced at the end of the evolution experiment. All contained three to six point mutations relative to LacI_wt_. All combinations of mutations were tested phenotypically, and the inverse mutants with the smallest number of mutations, which amounts to three for each, are described in this manuscript.

1. Poelwijk Frank J, de Vos Marjon GJ, Tans Sander J (2011) Tradeoffs and Optimality in the Evolution of Gene Regulation. Cell 146: 462-470.
